# 2-Amino-4-methyl­pyridinium trifluoro­acetate

**DOI:** 10.1107/S1600536810008202

**Published:** 2010-03-10

**Authors:** Madhukar Hemamalini, Hoong-Kun Fun

**Affiliations:** a X-ray Crystallography Unit, School of Physics, Universiti Sains Malaysia, 11800 USM, Penang, Malaysia

## Abstract

The asymmetric unit of the title compound, C_6_H_9_N_2_^+^·C_2_F_3_O_2_^−^, contains two independent 2-amino-4-methyl­pyridinium cations and two independent trifluoro­acetate anions. The F atoms of both anions are disordered over two sets of sites, with site occupancies of 0.50 (3) and 0.50 (3) in one of the anions, and 0.756 (9) and 0.244 (9) in the other. In the crystal, the cations and anions are linked into chains along the *b* axis by N—H⋯O hydrogen bonds and these chains are cross-linked by C—H⋯O hydrogen bonds, forming a two-dimensional network lying parallel to (101). The crystal structure is further stabilized by π–π inter­actions between the pyridinium rings [centroid–centroid distances = 3.5842 (13) and 3.5665 (16) Å].

## Related literature

For background to the chemistry of substituted pyridines, see: Pozharski *et al.* (1997 ▶); Katritzky *et al.* (1996 ▶). For related structures, see: Kvick & Noordik (1977 ▶); Shen *et al.* (2008 ▶); Hemamalini & Fun (2010 ▶). For trifluoro­acetic acid, see: Rodrigues *et al.* (2001 ▶). For details of hydrogen bonding, see: Jeffrey & Saenger (1991 ▶); Jeffrey (1997 ▶); Scheiner (1997 ▶). For hydrogen-bond motifs, see: Bernstein *et al.* (1995 ▶).
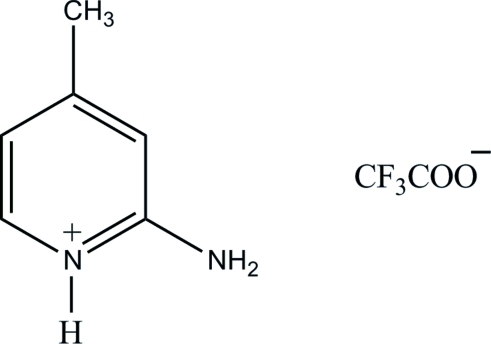


## Experimental

### 

#### Crystal data

C_6_H_9_N_2_^+^·C_2_F_3_O_2_^−^
*M* *_r_* = 222.17
Triclinic, 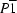

*a* = 8.5229 (2) Å
*b* = 11.0649 (3) Å
*c* = 11.6573 (3) Å
α = 81.208 (1)°
β = 72.199 (2)°
γ = 74.647 (1)°
*V* = 1006.26 (4) Å^3^
*Z* = 4
Mo *K*α radiation
μ = 0.14 mm^−1^
*T* = 296 K
0.56 × 0.19 × 0.08 mm

#### Data collection

Bruker SMART APEXII CCD area-detector diffractometer
Absorption correction: multi-scan (*SADABS*; Bruker, 2009 ▶) *T*_min_ = 0.925, *T*_max_ = 0.989
21533 measured reflections
5803 independent reflections
3405 reflections with *I* > 2σ(*I*)
*R*_int_ = 0.028

#### Refinement

*R*[*F* ^2^ > 2σ(*F* ^2^)] = 0.070
*wR*(*F* ^2^) = 0.187
*S* = 1.06
5803 reflections
357 parameters
114 restraints
H atoms treated by a mixture of independent and constrained refinement
Δρ_max_ = 0.25 e Å^−3^
Δρ_min_ = −0.26 e Å^−3^

### 

Data collection: *APEX2* (Bruker, 2009 ▶); cell refinement: *SAINT* (Bruker, 2009 ▶); data reduction: *SAINT*; program(s) used to solve structure: *SHELXTL* (Sheldrick, 2008 ▶); program(s) used to refine structure: *SHELXTL*; molecular graphics: *SHELXTL*; software used to prepare material for publication: *SHELXTL* and *PLATON* (Spek, 2009 ▶).

## Supplementary Material

Crystal structure: contains datablocks global, I. DOI: 10.1107/S1600536810008202/ci5038sup1.cif

Structure factors: contains datablocks I. DOI: 10.1107/S1600536810008202/ci5038Isup2.hkl

Additional supplementary materials:  crystallographic information; 3D view; checkCIF report

## Figures and Tables

**Table 1:** Hydrogen-bond geometry (Å, °)

*D*—H⋯*A*	*D*—H	H⋯*A*	*D*⋯*A*	*D*—H⋯*A*
N1*A*—H1*NA*⋯O2*A*^i^	0.89 (3)	1.85 (3)	2.740 (3)	173 (2)
N2*A*—H2*NA*⋯O1*A*^i^	0.85 (2)	2.02 (2)	2.871 (3)	176 (2)
N2*A*—H3*NA*⋯O2*B*^ii^	0.85 (2)	2.04 (2)	2.835 (3)	156 (2)
N1*B*—H1*NB*⋯O2*B*^ii^	0.87 (3)	1.86 (3)	2.734 (3)	175 (2)
N2*B*—H2*NB*⋯O1*B*^ii^	0.85 (2)	2.01 (2)	2.858 (3)	176 (3)
N2*B*—H3*NB*⋯O2*A*^iii^	0.83 (3)	2.04 (2)	2.848 (3)	164 (3)
C4*A*—H4*AA*⋯O1*B*^iv^	0.93	2.57	3.423 (3)	153
C6*B*—H6*BA*⋯O1*A*^v^	0.96	2.57	3.518 (5)	169

Symmetry codes: (i) 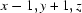; (ii) 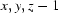; (iii) 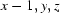; (iv) 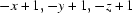; (v) 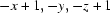.

**Fig. 1.**
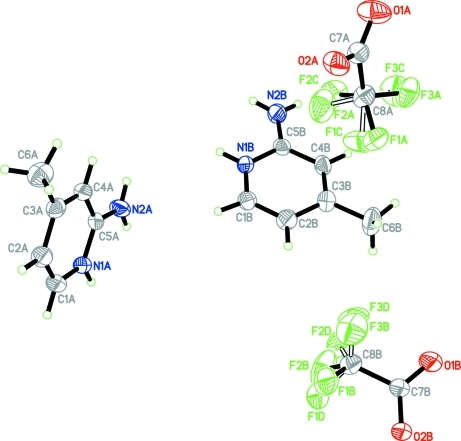
The asymmetric unit of the title compound. Displacement ellipsoids are drawn at the 30% probability level. All disorder components are shown.

**Fig. 2.**
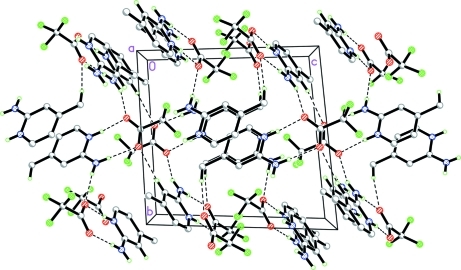
The crystal packing of the title compound, showing the hydrogen-bonded (dashed lines) network.

## supplementary crystallographic information

### Comment

Pyridine and its derivatives play an important role in heterocyclic chemistry (Pozharski *et al.*, 1997; Katritzky *et al.*, 1996). They are often involved in hydrogen-bond interactions (Jeffrey & Saenger, 1991; Jeffrey, 1997; Scheiner, 1997). Trifluoroacetic acid (TFA) is a very strong carboxylic acid, easily volatile, and used for protein purifications. Several trifluoroacetate salts and their crystal structures have been reported (Rodrigues *et al.*, 2001). The crystal structures of 2-amino-4-methylpyridine (Kvick & Noordik, 1977), 2-amino-4-methylpyridinium 4-aminobenzoate (Shen *et al.*, 2008) have also been reported. We have recently reported the crystal structure of 2-amino-4-methylpyridinium 4-nitrobenzoate (Hemamalini & Fun, 2010). In continuation of our studies of pyridinium derivatives, the crystal structure determination of the title compound has been undertaken.

The asymmetric unit of the title compound consists of two crystallographically independent 2-amino-4-methylpyridinium cations (A and B) and two trifluoroacetate anions (A and B) (Fig. 1).  Each 2-amino-4-methylpyridinium cation is planar, with a maximum deviation of 0.007 (3) Å for atom C6A in cation A and 0.011 (5) Å for atom C6B in cation B. The protonation of atoms N1A and N1B lead to a slight increase in C1A—N1A—C5A [122.3 (2)°] and C1B—N1B—C5B [121.7 (2)°] angles.

In the crystal packing (Fig. 2), the A (and B)-type 2-amino-4-methylpyridinium cations interact with the carboxylate groups of A (and B)-type trifluoroacetate anions through a pair of N—H···O  hydrogen bonds, forming an *R*_2_^2^(8) motif (Bernstein *et al.*, 1995). The cation-anion pairs are linked into a chain along the *b* axis by N—H···O  hydrogen bonds. The crystal structure is further stabilized by C—H···O (Table 1) hydrogen bonds and π–π interactions involving the N1A/C1A–C5A and N1A/C1A–C5A pyridine rings [centoid-to-centroid separation = 3.5842 (13) Å], and N1B/C1B–C5B and N1B/C1B–C5B rings [centoid-to-centroid separation = 3.5665 (16) Å].

### Experimental

To a hot methanol solution (20 ml) of 2-amino-4-methylpyridine (27 mg, Aldrich) was added a few drops of trifluoroacetic acid. The solution was warmed over a water bath for a few minutes. The resulting solution was allowed to cool slowly to room temperature. Crystals of the title compound appeared after a few days.

### Refinement

Atoms H1NA, H2NA, H3NA, H1NB, H2NB and H3NB were located in a difference Fourier map and refined; the N–H distances of the NH_2_ groups were restrained to be equal. The remaining H atoms were positioned geometrically [C–H = 0.93 or 0.96 Å] and were refined using a riding model, with *U*_iso_(H) = 1.2 or 1.5 *U*_eq_(C). A rotating group model was applied to the methyl groups. The F atoms of both anions are disordered over two positions, with site occupancies of 0.50 (3) and 0.50 (3) in one of the anions, and 0.756 (9) and 0.244 (9) in the other anion. In each anion, the C—F distances were restrained to be equal and the U^ij^ components of F atoms were restrained to an approximate isotropic behaviour.

### Crystal data

**Table:**

C_6_H_9_N_2_^+^·C_2_F_3_O_2_^−^	*Z* = 4
*M**_r_* = 222.17	*F*(000) = 456
Triclinic, *P*1	*D*_x_ = 1.467 Mg m^−^^3^
Hall symbol: -P 1	Mo *K*α radiation, λ = 0.71073 Å
*a* = 8.5229 (2) Å	Cell parameters from 4908 reflections
*b* = 11.0649 (3) Å	θ = 2.6–30.0°
*c* = 11.6573 (3) Å	µ = 0.14 mm^−^^1^
α = 81.208 (1)°	*T* = 296 K
β = 72.199 (2)°	Plate, colourless
γ = 74.647 (1)°	0.56 × 0.19 × 0.08 mm
*V* = 1006.26 (4) Å^3^	

### Data collection

**Table:**

Bruker SMART APEXII CCD area-detector diffractometer	5803 independent reflections
Radiation source: fine-focus sealed tube	3405 reflections with *I* > 2σ(*I*)
graphite	*R*_int_ = 0.028
φ and ω scans	θ_max_ = 30.0°, θ_min_ = 1.8°
Absorption correction: multi-scan (*SADABS*; Bruker, 2009)	*h* = −11→11
*T*_min_ = 0.925, *T*_max_ = 0.989	*k* = −15→15
21533 measured reflections	*l* = −16→16

### Refinement

**Table:**

Refinement on *F*^2^	Primary atom site location: structure-invariant direct methods
Least-squares matrix: full	Secondary atom site location: difference Fourier map
*R*[*F*^2^ > 2σ(*F*^2^)] = 0.070	Hydrogen site location: inferred from neighbouring sites
*wR*(*F*^2^) = 0.187	H atoms treated by a mixture of independent and constrained refinement
*S* = 1.06	*w* = 1/[σ^2^(*F*_o_^2^) + (0.0642*P*)^2^ + 0.3844*P*] where *P* = (*F*_o_^2^ + 2*F*_c_^2^)/3
5803 reflections	(Δ/σ)_max_ = 0.001
357 parameters	Δρ_max_ = 0.25 e Å^−^^3^
114 restraints	Δρ_min_ = −0.26 e Å^−^^3^

### Special details

**Table:**

Geometry. All s.u.'s (except the s.u. in the dihedral angle between two l.s. planes) are estimated using the full covariance matrix. The cell s.u.'s are taken into account individually in the estimation of s.u.'s in distances, angles and torsion angles; correlations between s.u.'s in cell parameters are only used when they are defined by crystal symmetry. An approximate (isotropic) treatment of cell s.u.'s is used for estimating s.u.'s involving l.s. planes.
Refinement. Refinement of F^2^ against ALL reflections. The weighted R-factor wR and goodness of fit S are based on F^2^, conventional R-factors R are based on F, with F set to zero for negative F^2^. The threshold expression of F^2^ > 2σ(F^2^) is used only for calculating R-factors(gt) etc. and is not relevant to the choice of reflections for refinement. R-factors based on F^2^ are statistically about twice as large as those based on F, and R- factors based on ALL data will be even larger.

### Fractional atomic coordinates and isotropic or equivalent isotropic displacement parameters (Å^2^)

**Table:**

	*x*	*y*	*z*	*U*_iso_*/*U*_eq_	Occ. (<1)
N1A	0.3581 (3)	0.99313 (18)	0.21338 (18)	0.0510 (5)	
N2A	0.2610 (3)	0.8369 (2)	0.1674 (2)	0.0672 (6)	
C1A	0.4811 (3)	1.0518 (2)	0.2081 (2)	0.0589 (6)	
H1AA	0.4538	1.1237	0.2490	0.071*	
C2A	0.6422 (3)	1.0074 (3)	0.1447 (2)	0.0605 (6)	
H2AA	0.7258	1.0485	0.1412	0.073*	
C3A	0.6831 (3)	0.8980 (2)	0.0835 (2)	0.0553 (6)	
C4A	0.5572 (3)	0.8401 (2)	0.0901 (2)	0.0548 (6)	
H4AA	0.5829	0.7678	0.0500	0.066*	
C5A	0.3900 (3)	0.8882 (2)	0.1564 (2)	0.0500 (6)	
C6A	0.8627 (4)	0.8466 (3)	0.0138 (3)	0.0779 (8)	
H6AA	0.8721	0.7662	−0.0125	0.117*	
H6AB	0.9358	0.8373	0.0645	0.117*	
H6AC	0.8951	0.9033	−0.0554	0.117*	
N1B	0.2268 (3)	0.49192 (19)	0.32553 (19)	0.0529 (5)	
N2B	0.1215 (4)	0.3395 (2)	0.2805 (2)	0.0708 (7)	
C1B	0.2776 (3)	0.5451 (3)	0.4013 (2)	0.0600 (7)	
H1BB	0.309 (4)	0.622 (3)	0.367 (3)	0.072 (8)*	
C2B	0.2732 (3)	0.4938 (3)	0.5135 (3)	0.0634 (7)	
H2BA	0.3074	0.5315	0.5648	0.076*	
C3B	0.2162 (3)	0.3817 (3)	0.5533 (2)	0.0612 (6)	
C4B	0.1643 (3)	0.3302 (2)	0.4769 (2)	0.0581 (6)	
H4BA	0.1248	0.2572	0.5027	0.070*	
C5B	0.1695 (3)	0.3855 (2)	0.3598 (2)	0.0508 (6)	
C6B	0.2136 (6)	0.3216 (4)	0.6779 (3)	0.0997 (12)	
H6BA	0.1825	0.2426	0.6877	0.149*	
H6BB	0.1326	0.3759	0.7365	0.149*	
H6BC	0.3240	0.3079	0.6894	0.149*	
F1A	0.7612 (16)	0.1532 (10)	0.5327 (7)	0.084 (2)	0.50 (3)
F2A	0.6477 (14)	0.1650 (13)	0.3757 (10)	0.102 (3)	0.50 (3)
F3A	0.6788 (15)	0.0165 (9)	0.5069 (8)	0.093 (3)	0.50 (3)
F1C	0.7539 (17)	0.1841 (16)	0.5129 (13)	0.110 (4)	0.50 (3)
F2C	0.6745 (18)	0.2046 (15)	0.3790 (11)	0.122 (4)	0.50 (3)
F3C	0.6439 (18)	0.0349 (13)	0.4851 (15)	0.117 (4)	0.50 (3)
O1A	0.9324 (3)	−0.0511 (2)	0.3165 (2)	0.0937 (7)	
O2A	1.0382 (2)	0.10073 (17)	0.34606 (19)	0.0712 (6)	
C7A	0.9242 (3)	0.0450 (2)	0.3593 (2)	0.0562 (6)	
C8A	0.7515 (3)	0.1063 (3)	0.4389 (2)	0.0636 (7)	
F1B	0.4287 (6)	0.6567 (4)	0.8679 (3)	0.0917 (13)	0.756 (9)
F2B	0.1802 (5)	0.6740 (7)	0.8575 (4)	0.136 (2)	0.756 (9)
F3B	0.3737 (6)	0.5082 (4)	0.8111 (4)	0.1062 (14)	0.756 (9)
F1D	0.310 (2)	0.7120 (8)	0.8858 (9)	0.101 (4)	0.244 (9)
F2D	0.1842 (14)	0.6008 (13)	0.8405 (11)	0.094 (4)	0.244 (9)
F3D	0.4324 (15)	0.5237 (15)	0.8247 (14)	0.122 (5)	0.244 (9)
O1B	0.2191 (3)	0.4371 (2)	1.03426 (19)	0.0899 (7)	
O2B	0.2503 (3)	0.60549 (17)	1.09745 (16)	0.0786 (6)	
C7B	0.2531 (3)	0.5397 (2)	1.0201 (2)	0.0566 (6)	
C8B	0.3048 (3)	0.5954 (3)	0.8898 (2)	0.0649 (7)	
H1NA	0.252 (4)	1.022 (2)	0.257 (2)	0.057 (7)*	
H2NA	0.161 (3)	0.869 (2)	0.209 (2)	0.062 (8)*	
H3NA	0.276 (4)	0.775 (2)	0.127 (2)	0.077 (9)*	
H1NB	0.238 (3)	0.524 (2)	0.251 (3)	0.058 (7)*	
H2NB	0.146 (4)	0.368 (3)	0.2065 (19)	0.079 (10)*	
H3NB	0.078 (3)	0.278 (2)	0.301 (3)	0.070 (9)*	

### Atomic displacement parameters (Å^2^)

**Table:**

	*U*^11^	*U*^22^	*U*^33^	*U*^12^	*U*^13^	*U*^23^
N1A	0.0562 (13)	0.0494 (11)	0.0466 (11)	−0.0169 (9)	−0.0063 (10)	−0.0099 (8)
N2A	0.0761 (17)	0.0585 (14)	0.0704 (15)	−0.0299 (13)	−0.0057 (13)	−0.0204 (12)
C1A	0.0699 (17)	0.0555 (14)	0.0587 (15)	−0.0232 (13)	−0.0167 (13)	−0.0136 (11)
C2A	0.0599 (16)	0.0667 (16)	0.0606 (15)	−0.0210 (13)	−0.0189 (13)	−0.0064 (12)
C3A	0.0599 (15)	0.0566 (14)	0.0451 (13)	−0.0056 (12)	−0.0168 (11)	−0.0005 (10)
C4A	0.0710 (16)	0.0439 (12)	0.0463 (13)	−0.0077 (11)	−0.0160 (12)	−0.0049 (10)
C5A	0.0684 (16)	0.0418 (11)	0.0407 (11)	−0.0174 (11)	−0.0143 (11)	0.0007 (9)
C6A	0.0636 (18)	0.084 (2)	0.076 (2)	−0.0031 (15)	−0.0145 (15)	−0.0103 (16)
N1B	0.0631 (13)	0.0487 (11)	0.0455 (11)	−0.0230 (10)	−0.0047 (10)	−0.0029 (9)
N2B	0.0989 (19)	0.0645 (15)	0.0620 (15)	−0.0456 (14)	−0.0189 (14)	−0.0039 (12)
C1B	0.0676 (17)	0.0579 (15)	0.0575 (15)	−0.0305 (13)	−0.0076 (12)	−0.0056 (12)
C2B	0.0653 (16)	0.0714 (17)	0.0621 (16)	−0.0281 (14)	−0.0186 (13)	−0.0066 (13)
C3B	0.0603 (15)	0.0629 (15)	0.0607 (15)	−0.0179 (12)	−0.0191 (12)	0.0055 (12)
C4B	0.0627 (15)	0.0490 (13)	0.0619 (15)	−0.0228 (12)	−0.0122 (12)	0.0048 (11)
C5B	0.0505 (13)	0.0451 (12)	0.0545 (14)	−0.0159 (10)	−0.0054 (10)	−0.0068 (10)
C6B	0.140 (3)	0.101 (3)	0.079 (2)	−0.054 (2)	−0.058 (2)	0.0303 (19)
F1A	0.096 (4)	0.091 (4)	0.054 (3)	−0.014 (3)	−0.010 (2)	−0.012 (3)
F2A	0.083 (4)	0.122 (5)	0.092 (4)	0.007 (4)	−0.037 (3)	−0.012 (4)
F3A	0.076 (4)	0.117 (4)	0.073 (4)	−0.038 (3)	0.000 (3)	0.015 (3)
F1C	0.101 (5)	0.114 (6)	0.127 (6)	−0.038 (4)	−0.010 (4)	−0.068 (5)
F2C	0.100 (5)	0.125 (6)	0.103 (5)	0.013 (4)	−0.025 (3)	0.023 (4)
F3C	0.080 (4)	0.131 (6)	0.146 (7)	−0.068 (4)	0.001 (4)	−0.024 (4)
O1A	0.0789 (14)	0.0947 (16)	0.1201 (19)	−0.0387 (12)	−0.0099 (13)	−0.0498 (14)
O2A	0.0621 (11)	0.0612 (11)	0.0907 (14)	−0.0314 (9)	−0.0031 (10)	−0.0147 (10)
C7A	0.0600 (15)	0.0598 (14)	0.0554 (14)	−0.0271 (12)	−0.0143 (12)	−0.0044 (11)
C8A	0.0613 (16)	0.0771 (19)	0.0573 (16)	−0.0277 (14)	−0.0137 (13)	−0.0049 (13)
F1B	0.098 (3)	0.100 (2)	0.0800 (17)	−0.053 (2)	−0.0076 (16)	0.0035 (15)
F2B	0.086 (2)	0.174 (4)	0.093 (2)	0.023 (3)	−0.0136 (18)	0.040 (3)
F3B	0.119 (3)	0.130 (3)	0.0665 (18)	−0.051 (2)	0.0142 (19)	−0.0440 (16)
F1D	0.146 (8)	0.084 (5)	0.079 (5)	−0.052 (5)	−0.025 (5)	0.008 (4)
F2D	0.105 (6)	0.113 (7)	0.084 (5)	−0.030 (5)	−0.062 (5)	0.012 (5)
F3D	0.078 (6)	0.148 (8)	0.112 (7)	−0.006 (5)	−0.002 (5)	−0.010 (6)
O1B	0.140 (2)	0.0703 (13)	0.0708 (13)	−0.0528 (14)	−0.0189 (13)	−0.0111 (10)
O2B	0.1372 (19)	0.0565 (11)	0.0532 (11)	−0.0412 (12)	−0.0259 (11)	−0.0054 (8)
C7B	0.0670 (16)	0.0506 (13)	0.0537 (14)	−0.0165 (12)	−0.0137 (12)	−0.0094 (11)
C8B	0.0657 (18)	0.0713 (18)	0.0547 (15)	−0.0147 (15)	−0.0106 (13)	−0.0108 (13)

### Geometric parameters (Å, °)

**Table:**

N1A—C5A	1.350 (3)	C2B—H2BA	0.93
N1A—C1A	1.354 (3)	C3B—C4B	1.356 (4)
N1A—H1NA	0.89 (3)	C3B—C6B	1.499 (4)
N2A—C5A	1.330 (3)	C4B—C5B	1.401 (3)
N2A—H2NA	0.855 (18)	C4B—H4BA	0.93
N2A—H3NA	0.850 (19)	C6B—H6BA	0.96
C1A—C2A	1.344 (4)	C6B—H6BB	0.96
C1A—H1AA	0.93	C6B—H6BC	0.96
C2A—C3A	1.408 (4)	F1A—C8A	1.314 (6)
C2A—H2AA	0.93	F2A—C8A	1.301 (6)
C3A—C4A	1.366 (3)	F3A—C8A	1.336 (6)
C3A—C6A	1.499 (4)	F1C—C8A	1.316 (6)
C4A—C5A	1.401 (3)	F2C—C8A	1.331 (6)
C4A—H4AA	0.93	F3C—C8A	1.306 (6)
C6A—H6AA	0.96	O1A—C7A	1.219 (3)
C6A—H6AB	0.96	O2A—C7A	1.243 (3)
C6A—H6AC	0.96	C7A—C8A	1.522 (4)
N1B—C5B	1.351 (3)	F1B—C8B	1.343 (4)
N1B—C1B	1.356 (3)	F2B—C8B	1.296 (4)
N1B—H1NB	0.88 (3)	F3B—C8B	1.326 (4)
N2B—C5B	1.329 (3)	F1D—C8B	1.297 (6)
N2B—H2NB	0.854 (19)	F2D—C8B	1.307 (7)
N2B—H3NB	0.830 (19)	F3D—C8B	1.269 (8)
C1B—C2B	1.338 (4)	O1B—C7B	1.220 (3)
C1B—H1BB	0.95 (3)	O2B—C7B	1.233 (3)
C2B—C3B	1.412 (4)	C7B—C8B	1.526 (4)
			
C5A—N1A—C1A	122.3 (2)	C4B—C3B—C2B	118.8 (2)
C5A—N1A—H1NA	116.6 (16)	C4B—C3B—C6B	121.1 (3)
C1A—N1A—H1NA	121.0 (16)	C2B—C3B—C6B	120.0 (3)
C5A—N2A—H2NA	121.2 (18)	C3B—C4B—C5B	120.9 (2)
C5A—N2A—H3NA	119 (2)	C3B—C4B—H4BA	119.5
H2NA—N2A—H3NA	119 (3)	C5B—C4B—H4BA	119.5
C2A—C1A—N1A	120.9 (2)	N2B—C5B—N1B	118.1 (2)
C2A—C1A—H1AA	119.5	N2B—C5B—C4B	123.9 (2)
N1A—C1A—H1AA	119.5	N1B—C5B—C4B	118.0 (2)
C1A—C2A—C3A	119.3 (2)	C3B—C6B—H6BA	109.5
C1A—C2A—H2AA	120.3	C3B—C6B—H6BB	109.5
C3A—C2A—H2AA	120.3	H6BA—C6B—H6BB	109.5
C4A—C3A—C2A	118.9 (2)	C3B—C6B—H6BC	109.5
C4A—C3A—C6A	121.2 (2)	H6BA—C6B—H6BC	109.5
C2A—C3A—C6A	119.9 (3)	H6BB—C6B—H6BC	109.5
C3A—C4A—C5A	120.8 (2)	O1A—C7A—O2A	128.6 (3)
C3A—C4A—H4AA	119.6	O1A—C7A—C8A	116.2 (2)
C5A—C4A—H4AA	119.6	O2A—C7A—C8A	115.2 (2)
N2A—C5A—N1A	117.9 (2)	F2A—C8A—F1A	122.3 (9)
N2A—C5A—C4A	124.3 (2)	F3C—C8A—F1C	114.7 (11)
N1A—C5A—C4A	117.7 (2)	F3C—C8A—F2C	106.7 (7)
C3A—C6A—H6AA	109.5	F1C—C8A—F2C	88.3 (16)
C3A—C6A—H6AB	109.5	F2A—C8A—F3A	104.7 (7)
H6AA—C6A—H6AB	109.5	F1A—C8A—F3A	93.2 (7)
C3A—C6A—H6AC	109.5	F2A—C8A—C7A	112.1 (6)
H6AA—C6A—H6AC	109.5	F1A—C8A—C7A	112.8 (6)
H6AB—C6A—H6AC	109.5	F3A—C8A—C7A	108.9 (5)
C5B—N1B—C1B	121.7 (2)	O1B—C7B—O2B	128.6 (3)
C5B—N1B—H1NB	118.8 (17)	O1B—C7B—C8B	116.2 (2)
C1B—N1B—H1NB	119.3 (16)	O2B—C7B—C8B	115.2 (2)
C5B—N2B—H2NB	120 (2)	F3D—C8B—F1D	116.9 (9)
C5B—N2B—H3NB	120 (2)	F3D—C8B—F2D	103.4 (9)
H2NB—N2B—H3NB	120 (3)	F1D—C8B—F2D	103.7 (7)
C2B—C1B—N1B	121.1 (2)	F2B—C8B—F3B	108.5 (4)
C2B—C1B—H1BB	125.9 (17)	F2B—C8B—F1B	106.5 (4)
N1B—C1B—H1BB	112.9 (17)	F3B—C8B—F1B	101.8 (3)
C1B—C2B—C3B	119.4 (2)	F2B—C8B—C7B	113.0 (3)
C1B—C2B—H2BA	120.3	F3B—C8B—C7B	112.8 (3)
C3B—C2B—H2BA	120.3	F1B—C8B—C7B	113.5 (2)
			
C5A—N1A—C1A—C2A	0.2 (4)	O1A—C7A—C8A—F3C	20.8 (11)
N1A—C1A—C2A—C3A	−0.3 (4)	O2A—C7A—C8A—F3C	−159.7 (11)
C1A—C2A—C3A—C4A	0.2 (4)	O1A—C7A—C8A—F1A	141.7 (6)
C1A—C2A—C3A—C6A	−179.2 (3)	O2A—C7A—C8A—F1A	−38.8 (6)
C2A—C3A—C4A—C5A	0.0 (4)	O1A—C7A—C8A—F1C	159.8 (10)
C6A—C3A—C4A—C5A	179.4 (2)	O2A—C7A—C8A—F1C	−20.6 (10)
C1A—N1A—C5A—N2A	179.6 (2)	O1A—C7A—C8A—F2C	−101.8 (11)
C1A—N1A—C5A—C4A	0.0 (3)	O2A—C7A—C8A—F2C	77.7 (11)
C3A—C4A—C5A—N2A	−179.7 (2)	O1A—C7A—C8A—F3A	39.7 (6)
C3A—C4A—C5A—N1A	−0.1 (3)	O2A—C7A—C8A—F3A	−140.8 (6)
C5B—N1B—C1B—C2B	0.1 (4)	O1B—C7B—C8B—F3D	−57.7 (10)
N1B—C1B—C2B—C3B	0.6 (4)	O2B—C7B—C8B—F3D	122.7 (10)
C1B—C2B—C3B—C4B	−1.2 (4)	O1B—C7B—C8B—F2B	96.6 (5)
C1B—C2B—C3B—C6B	178.8 (3)	O2B—C7B—C8B—F2B	−83.0 (5)
C2B—C3B—C4B—C5B	1.1 (4)	O1B—C7B—C8B—F1D	169.3 (10)
C6B—C3B—C4B—C5B	−179.0 (3)	O2B—C7B—C8B—F1D	−10.3 (10)
C1B—N1B—C5B—N2B	179.7 (3)	O1B—C7B—C8B—F2D	55.9 (8)
C1B—N1B—C5B—C4B	−0.3 (4)	O2B—C7B—C8B—F2D	−123.7 (7)
C3B—C4B—C5B—N2B	179.6 (3)	O1B—C7B—C8B—F3B	−26.9 (4)
C3B—C4B—C5B—N1B	−0.3 (4)	O2B—C7B—C8B—F3B	153.5 (3)
O1A—C7A—C8A—F2A	−75.6 (8)	O1B—C7B—C8B—F1B	−142.0 (3)
O2A—C7A—C8A—F2A	103.9 (8)	O2B—C7B—C8B—F1B	38.4 (4)

### Hydrogen-bond geometry (Å, °)

**Table:**

*D*—H···*A*	*D*—H	H···*A*	*D*···*A*	*D*—H···*A*
N1A—H1NA···O2A^i^	0.89 (3)	1.85 (3)	2.740 (3)	173 (2)
N2A—H2NA···O1A^i^	0.85 (2)	2.02 (2)	2.871 (3)	176 (2)
N2A—H3NA···O2B^ii^	0.85 (2)	2.04 (2)	2.835 (3)	156 (2)
N1B—H1NB···O2B^ii^	0.87 (3)	1.86 (3)	2.734 (3)	175 (2)
N2B—H2NB···O1B^ii^	0.85 (2)	2.01 (2)	2.858 (3)	176 (3)
N2B—H3NB···O2A^iii^	0.83 (3)	2.04 (2)	2.848 (3)	164 (3)
C4A—H4AA···O1B^iv^	0.93	2.57	3.423 (3)	153
C6B—H6BA···O1A^v^	0.96	2.57	3.518 (5)	169

Symmetry codes: (i) *x*−1, *y*+1, *z*; (ii) *x*, *y*, *z*−1; (iii) *x*−1, *y*, *z*; (iv) −*x*+1, −*y*+1, −*z*+1; (v) −*x*+1, −*y*, −*z*+1.
